# Flavivirus-induced antibody cross-reactivity

**DOI:** 10.1099/vir.0.031641-0

**Published:** 2011-12

**Authors:** Karen L. Mansfield, Daniel L. Horton, Nicholas Johnson, Li Li, Alan D. T. Barrett, Derek J. Smith, Sareen E. Galbraith, Tom Solomon, Anthony R. Fooks

**Affiliations:** 1 Wildlife Zoonoses and Vector-borne Diseases Research Group, Animal Health and Veterinary Laboratories Agency, Woodham Lane, New Haw, Addlestone, Surrey KT15 3NB, UK; 2 Brain Infections Group, University of Liverpool, UK; 3 Department of Zoology, University of Cambridge, Downing Street, Cambridge CB2 3EJ, UK; 4 Department of Pathology, University of Texas Medical Branch, Galveston, Texas, USA; 5 National Centre for Zoonoses Research, University of Liverpool, UK

## Abstract

Dengue viruses (DENV) cause countless human deaths each year, whilst West Nile virus (WNV) has re-emerged as an important human pathogen. There are currently no WNV or DENV vaccines licensed for human use, yet vaccines exist against other flaviviruses. To investigate flavivirus cross-reactivity, sera from a human cohort with a history of vaccination against tick-borne encephalitis virus (TBEV), Japanese encephalitis virus (JEV) and yellow fever virus (YFV) were tested for antibodies by plaque reduction neutralization test. Neutralization of louping ill virus (LIV) occurred, but no significant neutralization of Murray Valley encephalitis virus was observed. Sera from some individuals vaccinated against TBEV and JEV neutralized WNV, which was enhanced by YFV vaccination in some recipients. Similarly, some individuals neutralized DENV-2, but this was not significantly influenced by YFV vaccination. Antigenic cartography techniques were used to generate a geometric illustration of the neutralization titres of selected sera against WNV, TBEV, JEV, LIV, YFV and DENV-2. This demonstrated the individual variation in antibody responses. Most sera had detectable titres against LIV and some had titres against WNV and DENV-2. Generally, LIV titres were similar to titres against TBEV, confirming the close antigenic relationship between TBEV and LIV. JEV was also antigenically closer to TBEV than WNV, using these sera. The use of sera from individuals vaccinated against multiple pathogens is unique relative to previous applications of antigenic cartography techniques. It is evident from these data that notable differences exist between amino acid sequence identity and mapped antigenic relationships within the family *Flaviviridae*.

## Introduction

Within the family *Flaviviridae*, the genus *Flavivirus* comprises tick-borne, mosquito-borne and no-known-vector viruses, and virus species are further placed into groups with shared antigenic cross-reactivity (Porterfield, 1980). West Nile virus (WNV) belongs to the Japanese encephalitis virus (JEV) serocomplex along with viruses including JEV, Murray Valley encephalitis virus (MVEV) and St. Louis encephalitis virus. Other serocomplexes include yellow fever virus (YFV), Dengue virus (DENV) and tick-borne encephalitis virus (TBEV), which contains three subtypes of TBEV and louping ill virus (LIV) (Porterfield, 1980; Calisher *et al.*, 1989). This classification has largely been supported by genomic phylogeny (Gaunt *et al.*, 2001; Grard *et al.*, 2007).

A number of flaviviruses constitute a significant threat to global health. DENV infection causes around 21 000 human deaths annually, and it is estimated that at least 120 countries have endemic DENV transmission (Thomas & Endy, 2011), whilst in recent years, WNV has become more prominent as a zoonotic agent, particularly in North America where the virus first emerged in 1999 and rapidly spread across the continent (Nash *et al.*, 2001). WNV has now emerged in a number of European countries, particularly around the Mediterranean basin, where infections in humans, horses and birds have been reported (Hubálek *et al.*, 1999; Murgue *et al.*, 2001; Autorino *et al.*, 2002; Bakonyi *et al.*, 2006; Krisztalovics *et al.*, 2008; Rizzo *et al.*, 2009). In particular, a substantial number of human infections have recently been detected in Greece (Papa *et al.*, 2010), which were preceded by the detection of antibodies to WNV in earlier human and animal seroprevalence studies. This suggests that WNV had been circulating previously, and certain conditions had favoured increased numbers of *Culex* mosquitoes, which resulted in human WNV cases. As the European climate continues to change, this could occur elsewhere in Europe.

There are no licensed vaccines approved for use in humans against WNV (Kramer *et al.*, 2008) or DENV (Thomas & Endy, 2011). However, human vaccines are available against other flaviviruses, including TBEV, JEV and YFV. Cross-reactivity of sera raised against one flavivirus recognizing another flavivirus has been well documented (Calisher *et al.*, 1989). Studies with mAbs have identified cross-reactive sites on the envelope glycoproteins of different flavivirus species (Peiris *et al.*, 1982; Takegami *et al.*, 1982; Kimura-Kuroda & Yasui, 1986; Heinz *et al.*, 1983; Schlesinger & Brandriss, 1983; Beasley & Barrett, 2002), but relationships within and between serocomplexes are inconsistent (Calisher *et al.*, 1989). One consequence of flavivirus cross-reactivity is the occurrence of false-positive results (Hirota *et al.*, 2010), yet cross-reactivity can lead to cross-protection. Pre-existing vaccine-induced immunity to TBEV has been shown to enhance the neutralizing antibody response following vaccination with an inactivated JEV vaccine candidate (Schuller *et al.*, 2008), whilst separate studies in hamsters have demonstrated that prior inoculation with one flavivirus lead to a reduction in severity of subsequent challenge with a different flavivirus (Tesh *et al.*, 2002; Bosco-Lauth *et al.*, 2011).

A panel of sera was obtained from a human cohort vaccinated against TBEV, JEV and YFV (*n* = 28), and the neutralization profile assessed against a range of flaviviruses. Sera were tested by plaque reduction neutralization test (PRNT), and assessment was made both within, and between, serocomplex groups. Using antigenic cartography techniques, a geometric interpretation of the titres of selected human sera against WNV, TBEV, JEV, LIV, YFV (two strains) and DENV-2 was made. This analysis is unique relative to previous uses of antigenic cartography for influenza, rabies and enterovirus (Smith *et al.*, 2004; de Jong *et al.*, 2007; Russell *et al.*, 2008; Huang *et al.*, 2009; Garten *et al.*, 2009; Horton *et al.*, 2010), enabling high resolution quantitative analysis and visualization of antigenic relationships of flaviviruses.

## Results

### Neutralizing antibody response to vaccination against TBEV, JEV and YFV

**Fig. 1.**
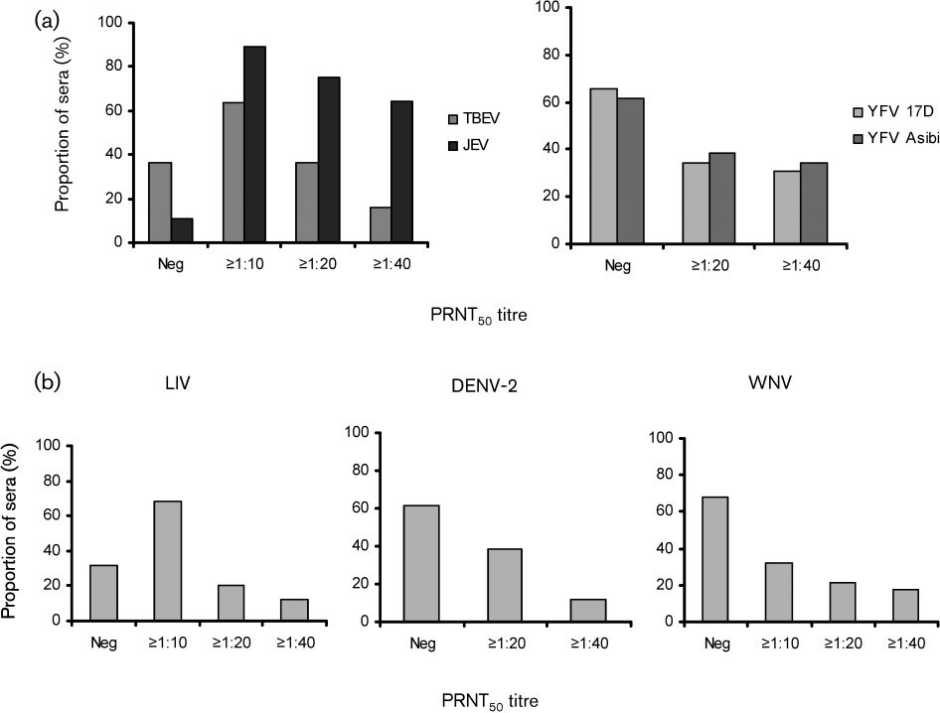
(a) Seroconversion following vaccination against TBEV (*n* = 25) and JEV (*n* = 28) (left panel), and YFV (*n* = 26) (right panel; strain 17D and strain Asibi). Proportion of sera (% sera) achieving a neutralizing antibody titre of ≥1 : 10, ≥1 : 20 or ≥1 : 40, or no neutralization (Neg), as determined by PRNT_50_. (b) Cross-neutralization of LIV (*n* = 25), WNV (*n* = 28) and DENV-2 (*n* = 26) (left, centre and right panels, respectively). Proportion of sera (% sera) achieving a neutralizing antibody titre of ≥1 : 10, ≥1 : 20 and ≥1 : 40, or no neutralization, as determined by PRNT_50_.

All the neutralization results for the sera obtained from the flavivirus-vaccinated human cohort are provided in Supplementary Table S1 (available in JGV Online). When tested by TBEV-specific PRNT, 64 % of the samples tested (*n* = 25) achieved a >50 % reduction in plaque numbers (PRNT_50_) titre of at least 1 : 10, 36 % achieved a PRNT_50_ titre of at least 1 : 20 and 16 % achieved a PRNT_50_ titre of at least 1 : 40 (Fig. 1a, left panel).

Neutralizing antibody to JEV was observed in 89.3 % of the samples tested (*n* = 28) as assessed by JEV-specific PRNT, where a PRNT_50_ titre of at least 1 : 10 was achieved. Indeed, 64.3 % of the samples achieved a PRNT_50_ titre of at least 1 : 40 (Fig. 1a, left panel).

Neutralizing antibody response against the 17D and Asibi strains of YFV was determined by YFV-specific PRNT, where PRNT_50_ titres of at least 1 : 20 were achieved by 34.6 and 38.5 % of the samples tested (*n* = 26) against 17D and Asibi, respectively (Fig. 1a, right panel).

### Effect of gender on neutralizing antibody response

Gender of participants appeared to have no significant effect on neutralizing antibody titres (data not shown) (Student’s *t*-test: *P* = 0.41, 0.06, 0.34, 0.55, 0.37, 0.73 and 0.97 for TBEV, JEV, LIV, WNV, YFV 17D, YFV Asibi and DENV-2, respectively).

### Cross-neutralization of LIV

A majority of individuals were able to neutralize LIV, with 68 % of samples demonstrating a PRNT_50_ titre of at least 1 : 10 and a further 20 % demonstrating a titre of 1 : 20 or higher (Fig. 1b, left panel). One individual achieved a titre of 1 : 1280.

The Spearman’s rank correlation coefficient (0.674) between the titres of LIV and TBEV PRNT_50_ was highly significant (*P*<0.001).

### Cross-neutralization of MVEV

None of the samples from the cohort demonstrated detectable levels of neutralization of MVEV by PRNT_50_ (Supplementary Table S1). However, minimal neutralization was observed at a serum dilution of 1 : 10 for some participants, but this was not sufficient to give a positive PRNT_50_ titre, and was not related to vaccination against YFV in those individuals (Student’s *t*-test, *P* = 0.622).

### Cross-neutralization of DENV-2

Of the samples tested (*n* = 26), 38.5 % achieved a PRNT_50_ titre of at least 1 : 20, with 11.5 % achieving a titre of at least 1 : 40 (Fig. 1b, centre panel). Spearman’s rank correlation analysis provided no evidence of an association between titres against DENV-2 and either JEV, YFV 17D or YFV Asibi (*P* = 0.530, 0.346 and 0.117, respectively). Furthermore, the titres from YFV-vaccinated individuals were not significantly different from individuals who were not vaccinated against YFV (Kruskall–Wallis test, *P* = 0.070), indicating that any effect of vaccination against YFV on the likelihood of neutralization of DENV-2, could not be detected using these data.

### Cross-neutralization of WNV

**Fig. 2.**
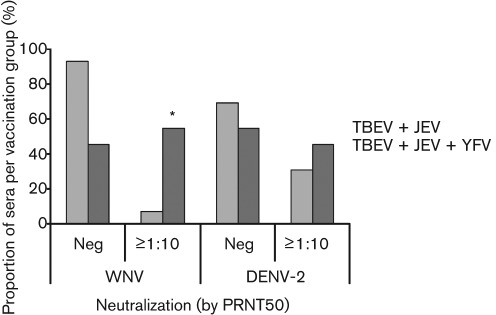
Effect of vaccination against YFV on WNV and DENV-2 PRNT_50_ titre: percentage of samples per vaccination group (TBEV+JEV vaccination *n* = 14; TBEV+JEV+YFV vaccination *n* = 11) achieving either a neutralizing antibody titre of at least 1 : 10 by PRNT_50_, or no neutralization (Neg), against WNV or DENV-2. Samples with uncertain YFV were excluded from the analysis. Statistical significance between the two vaccination groups was calculated by Kruskall–Wallis test (*P* = 0.002), and is denoted by *.

Cross-neutralization of WNV by PRNT (achieving a PRNT_50_ titre of at least 1 : 10) was observed in nine individuals (32.1 % of the sample population), where six individuals (21.4 %) recorded a PRNT_50_ titre of 1 : 20 or higher (Fig. 1b, right panel). The Spearman’s rank correlation coefficient (0.490) between PRNT_50_ titres against WNV and YFV Asibi indicate a significant positive association (*P* = 0.017). Similarly, the Spearman’s rank correlation coefficient (0.386) between PRNT_50_ titres against WNV and YFV 17D indicate a positive association that is just significant (*P* = 0.047). Individuals who had been vaccinated against YFV as well as TBEV and JEV were more likely to neutralize WNV (achieving a PRNT_50_ titre of at least 1 : 10) than individuals who had only been vaccinated against TBEV and JEV (Fig. 2). Three individuals were unable to confirm whether they had received YFV vaccination. Of the individuals who had been vaccinated against TBEV and JEV only (*n* = 14), only 7.1 % were able to neutralize WNV, achieving a PRNT_50_ titre of at least 1 : 10. In comparison, of the individuals who had received an additional vaccination against YFV (*n* = 11), 54.5 % demonstrated neutralization of WNV, achieving a PRNT_50_ titre of at least 1 : 10. The titres of YFV-vaccinated individuals were significantly higher than those from unvaccinated individuals (Kruskall–Wallis test, *P* = 0.002), confirming that people vaccinated against YFV were more likely to neutralize WNV than those not vaccinated against YFV. This result confirmed that vaccination status appeared to influence the ability to neutralize WNV. From this study, the effect of JEV vaccination on the ability to neutralize WNV is unclear, since the majority of individuals were known to have received the vaccination. The proportion of sera with a JEV PRNT_50_ titre of at least 1 : 10 for the WNV PRNT-negative samples (84.2 %) was not significantly different from that for the WNV PRNT-positive samples (100 %) by Fisher’s exact test (*P* = 0.530), whereas the Spearman’s rank correlation coefficient (0.437) between the titres of the two tests was significant (*P* = 0.021).

Results for all PRNT data from individuals that neutralized WNV are detailed in Table 1.

**Table 1.** Summary of PRNT results for sera that demonstrated neutralization of WNV nd, Not determined.

YFV vaccination	Neutralizing antibody titre (PRNT_50_)							
YFV (17D)	YFV (Asibi)	TBEV	JEV	LIV	DENV-2	WNV	MVEV
No	Neg	Neg	1 : 40	1 : 160	1 : 10	Neg	1 : 20	Neg
Yes	Neg	Neg	Neg	1 : 40	Neg	Neg	1 : 10	Neg
Yes	1 : 40	1 : 80	1 : 20	1 : 40	1 : 10	1 : 20	1 : 40	Neg
Yes	1 : 80	1 : 80	Neg	1 : 10	Neg	1 : 20	1 : 10	Neg
Yes	1 : 20	1 : 20	Neg	1 : 160	Neg	1 : 20	1 : 160	Neg
Yes	1 : 40	1 : 80	1 : 160	1 : 80	1 : 40	1 : 80	1 : 80	Neg
Yes	1 : 80	1 : 160	nd	1 : 160	1 : 10	1 : 20	1 : 80	Neg
Unknown	nd	nd	nd	1 : 80	Neg	nd	1 : 10	Neg
Unknown	Neg	1 : 40	1 : 1280	1 : 160	1 : 1280	1 : 40	1 : 40	Neg

### Visualization of the neutralization data using antigenic cartography techniques

**Fig. 3.**
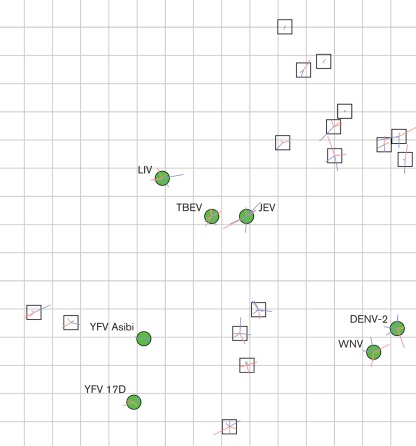
Geometric illustration of selected serum neutralization titres against TBEV, LIV, JEV, WNV, YFV (two strains) and DENV-2 made using antigenic cartography techniques. Sera are represented by white squares, and viruses are represented by green circles. Coloured error lines are shown for each virus and serum. The length of the error line is proportional to the difference between distance on the figure and the target distance; the colour indicates the direction of the difference (blue = positive difference, i.e. the distance on the figure is more than the target distance; red = negative).

Antigenic cartography has provided a novel means of visualizing and quantifying the relationship between serum titres and closely related antigens to which they react. The geometric interpretation of the neutralization titres is illustrated in Fig. 3, demonstrating the antigenic relationships among TBEV, JEV, LIV, WNV, YFV and DENV-2 as determined by these sera. The dispersal of the sera on the map illustrates the differences in each participant’s cross-neutralization responses. Despite being from volunteers only vaccinated against TBEV, JEV and in some cases YFV, most sera had detectable titres against LIV and some had titres against WNV and DENV-2. In most cases the LIV titres were similar to the titre against TBEV, confirming the close antigenic relationship between TBEV and LIV. However, using these sera, JEV was also antigenically closer to TBEV than WNV, whilst WNV was in close proximity to DENV-2. Error lines are shown for each virus and serum. The length of the error lines is proportional to the difference between the distance on the figure and the target distance, and the colour indicates the direction of the difference (blue = positive difference, i.e. the distance on the figure is more than the target distance; red = negative). The error lines are short, indicating that the errors associated with the positions of the points were small.

MVEV does not appear on the map, as serum cross-neutralization was not sufficiently robust to position the virus relative to the others analysed.

## Discussion

Both innate and adaptive immune responses are necessary in order to control flavivirus infection, with the initiation of an early antibody response providing a critical element of virus control (Diamond *et al.*, 2003a, b). The flavivirus envelope glycoprotein is the principal antigen responsible for eliciting a neutralizing antibody response, although antibodies specific for the prM and NS1 proteins have also been detected (Pincus *et al.*, 1992; Colombage *et al.*, 1998; Shu *et al.*, 2000; Chung *et al.*, 2007). Indeed, the resistance of WNV to antibody-mediated neutralization has been directly correlated with mutations in the epitopes of envelope protein domain III (ED3) (Maillard *et al.*, 2008), and a potential role for ED3 in cross-protection among flaviviruses has been suggested following studies in mice (Li *et al.*, 2011).

Antibody-mediated immunity is therefore considered a major modality of protection against members of the flavivirus serocomplex and is a useful and measurable correlate of vaccine-induced immunity (Vaughan *et al.*, 2010). However, there are clearly contradictions and paradoxes in our current understanding of flavivirus antigenic relationships that have direct consequences for predicting protection and for serodiagnosis. Antigenic cartography has been applied to other pathogens, allowing high resolution quantitative analyses and visualizations of antigenic relationships, using sera represented as a single point thus allowing robust and repeatable maps to be made (Smith *et al.*, 2004; de Jong *et al.*, 2007; Russell *et al.*, 2008; Huang *et al.*, 2009; Garten *et al.*, 2009; Horton *et al.*, 2010). The analysed sera were from volunteers vaccinated with multiple pathogens, whereas previous applications of antigenic cartography have used sera produced using either infection or vaccination with a single pathogen. An assumption in current antigenic cartography methods is that the position of the sera on the maps can be represented by a single point. However, this assumption will only hold if the polyclonal sera (which will typically include populations of antibodies which bind to different antigenic components of the virus, and with differing affinities) can be represented as one ‘centre of action’. In previous studies, this assumption holds to the first order for sera that have been raised to a single antigen (Smith *et al.*, 2004; de Jong *et al.*, 2007; Russell *et al.*, 2008; Huang *et al.*, 2009; Horton *et al.*, 2010). The single point assumption would not be expected to hold for multi-infection sera, as the sera will contain an even more diverse population of antibodies than single-infection polyclonal sera, and the assumption of sera being adequately represented by a single point is even less likely to be satisfied. Despite the nature of the sera in the current study, the error associated with the position of the sera on Fig. 3 is small, the maps are robust and repeatable, and this geometric interpretation of the data is reliable. An important caveat when interpreting these data is that it does not necessarily reflect the true antigenic distances among these viruses; instead it represents how these particular sera relate to the antigens. For example, given sufficient sera from individuals vaccinated using a combined measles, mumps and rubella vaccine (M-M-R), it may be possible to make a geometric interpretation of the relationships between the sera. However, this does not imply that measles, mumps and rubella viruses are antigenically related. Future analyses using different sera would be expected to show different relationships. A thorough quantitative analysis of antigenic variation among and within flavivirus serocomplexes using single-infection sera is required, in order to better understand the complex antigenic relationships among the flaviviruses.

One advantage of antigenic cartography is that it is minimally dependent on individual variations in serological response compared with other interpretations of antigenic data. The cross-reactivities of the sera to the viruses in the study, and the relationships of the sera to each other, can be reliably interpreted from Fig. 3. In these volunteers, vaccination against JEV has failed to produce sufficient broad cross-neutralization either within the JEV serocomplex to generate high titres against WNV, nor against DENV-2 which is in a different serocomplex. Hence WNV and DENV-2 are much more antigenically distant from JEV than TBEV is from JEV. However, we cannot exclude the possibility that formalin inactivation of the killed JEV vaccine denatured epitopes that cross-react with WNV. Furthermore, the proximity of JEV to TBEV, and WNV to DENV-2 would not be expected considering their genetic relationships, but is likely to be related to these sera coming from volunteers vaccinated with both TBEV and JEV. Antigenic relationships measured using sera raised against alternative viruses are likely to be different. We can also draw conclusions regarding the positions of the sera. Despite having similar vaccination regimes, sera differ greatly in the breadth and focus of response. This is apparent from the raw titres, but is easily visualized in Fig. 3 where the sera are well distributed, with the response of some individuals biased towards the TBEV serocomplex and the response of others biased towards the YFV serocomplex.

We have demonstrated that human vaccination against TBEV and JEV elicited a limited antibody response capable of neutralizing lineage 1 WNV, which is the predominant lineage circulating in Europe. Although this study did not control for some variables such as the age of the subjects, or the time between YFV vaccination and blood sampling, the results suggest that additional vaccination against YFV enhanced the ability to neutralize WNV. This conclusion is at variance with a previous study that concluded that JEV vaccination was more effective than YFV vaccination for neutralization of WNV (Yamshchikov *et al.*, 2005). Our observation is surprising as YFV and WNV are genetically distinct and belong to different serocomplex groups, a feature corroborated by the antigenic cartographical analysis (Fig. 3). However, current vaccines against YFV are live-attenuated rather than inactivated, and are likely to be more immunogenic, stimulating a broader spectrum of responses and inducing a strong humoral immune response. Both the TBEV and the JEV vaccines used in this study were formalin-inactivated preparations.

Most individuals vaccinated against TBEV elicited a measurable titre to LIV. There is currently no human vaccine available for LIV but the results presented in this study suggest that vaccination against TBEV may offer partial protection against infection with LIV. Indeed, a TBEV vaccine based upon the European prototype strain Neudoerfl has recently been shown to induce cross-reactive antibodies in humans, against both other TBEV subtypes and more distantly related viruses, such as Omsk hemorrhagic fever virus (Orlinger *et al.*, 2011). Conversely, despite the fact that MVEV is genetically and apparently antigenically closely related to JEV according to previous studies (Calisher *et al.*, 1989), we observed no significant neutralization of MVEV following vaccination with an inactivated whole-virus JEV vaccine. These data support an earlier study, where sera from mice vaccinated with a licensed inactivated JEV vaccine (JE-VAX) demonstrated negligible neutralization of MVEV (Lobigs *et al.*, 2009).

This study has demonstrated that individuals who had received vaccinations against TBEV, JEV and YFV were able to neutralize LIV and to a lesser degree lineage 1 WNV and DENV-2, but not MVEV. However, the majority of individuals did not develop neutralizing antibodies against WNV or DENV-2. These data suggest that this vaccination regimen cannot be recommended to provide consistent protective immune responses against WNV or DENV, and emphasizes the need for development of virus-specific vaccines for use in high-risk groups of infection or severe disease. However, these data were obtained using specific virus strains for PRNT, and the use of alternative virus strains may lead to some degree of variation in the results. Furthermore, the presence of neutralizing antibodies against the virus strains used in this study does not guarantee protection against alternative strains and currently circulating viruses.

Utilization and manipulation of the cross-reactive properties of flaviviruses have the potential to assist the development of effective broad-spectrum human vaccines against WNV and other existing and emerging flaviviruses. ChimeriVax technology has advantageously taken the cross-reactive properties of flaviviruses one step further in the development of vaccine candidates (Arroyo *et al.*, 2004; Lobigs *et al.*, 2009). Ultimately, the development of a broad-spectrum chimeric vaccine with components tailored to individual viruses would be of benefit in countries where a number of different flaviviruses co-circulate.

## Methods

### 

#### Samples.

A cohort of 28 individuals (all of white Caucasian ethnic origin) was established, with a history of vaccination against flaviviruses. This was primarily based on occupational vaccination for TBEV (TicoVac, Baxter) and JEV [Japanese Encephalitis (JE) Vaccine; GCC, Green Cross Corporation]. Blood samples were taken on completion of the full course of JEV vaccination (which comprises three doses on days 0, 7 and 28), and after the second vaccination in the course of TBEV vaccination (which is given 1–3 months after the first vaccination). The mean number of days (with sem) between vaccination and blood sampling was 22.5 days (sem 10.3 days) and 75.2 days (sem 32.4 days) for TBEV and JEV vaccinations, respectively. There was one individual within the sample population who had previously completed the full course of three vaccinations against TBEV, and had also received two booster vaccinations. Similarly, they had received a JEV booster nearly 2 years after completing the initial course of vaccinations. The time between last vaccination and blood sampling for this individual was 20 months and 22 months for TBEV and JEV vaccinations, respectively. Furthermore, within the cohort were 11 individuals who had previously received vaccination for YFV (manufacturer not recorded), and the mean number of years between vaccination and blood sampling was 9.2 years (sem 5.1 years). Sera were separated from whole blood samples and heat inactivated at 56 °C for 30 min.

#### Viruses.

The viruses selected for use in PRNT included lineage 1 WNV, European TBEV, LIV, JEV and MVEV, YFV (two strains) and DENV-2. Specific details on the origin of each virus are provided in Supplementary Table S2 (available in JGV Online).

#### PRNT.

PRNT was used to determine cross-neutralization against WNV, LIV, MVEV or DENV-2, and to demonstrate seroconversion following vaccination against TBEV, JEV or YFV (Office Internationale des Epizooties (OIE), 2008). Briefly, a twofold dilution series of serum was incubated with an equal volume of virus for 30–60 min at 37 °C/5 % CO_2_. Serum dilutions were incubated with Vero C1008 cells for 30–60 min at 37 °C/5 % CO_2_ along with wells set up with virus-only and media-only controls. Following overlay, plates were incubated at 37 °C/5 % CO_2_ for 2 (MVEV), 3 (WNV), 4 (YFV and DENV-2), 5 (JEV) or 6 days (TBEV/LIV). Following fixation and staining, plaques were counted in each well, and titre calculated according to the percentage reduction in plaques in comparison with the virus control wells (PRNT_50_: >50 % reduction in plaque numbers). Serum samples giving titres ≥1 : 10 were considered positive.

#### Visualization of neutralization data using antigenic cartography techniques.

Using techniques described previously (Smith *et al.*, 2004; Horton *et al.*, 2010) we illustrated the relationships of TBEV, LIV, JEV, WNV, YFV (two strains) and DENV-2 according to the titres of selected sera (*n* = 17) that neutralized at least three viruses (<10 is equivalent to negative). Briefly, this first required deriving a target distance from each serum to each of the seven viruses. This was done by taking the logarithm_2_ of all the reciprocal titres, then calculating the difference between the titre against one particular virus and the maximum titre achieved by that serum (against any virus) or 1280, whichever was higher. Neutralization titres (expressed as ED_50_ = reciprocal 50 % end point dilutions) and target distances used for Fig. 3 are shown in Supplementary Table S3 (available in JGV Online). Thus, the target distance between a serum and virus depends upon the titre against that virus. The target distance is relative to a fixed higher titre and therefore the higher the titre of the serum against a virus, the shorter the target distance between that serum and virus. Antigenic cartography (Smith *et al.*, 2004) was then used to optimize the positions of the viruses and sera relative to each other such that the distance between sera and viruses on the illustration was as close as possible to the target distance (minimizing the sum-squared error between the distance on the illustration and the target distance). Each virus is therefore positioned by multiple sera, and the sera themselves are also positioned by their distances to the viruses. To increase the likelihood of obtaining the best match to the target distances, multiple random restart optimizations (100) were undertaken as in previous analyses, creating multiple maps that were ranked in order of total error and quantitatively compared for self-consistency. As in antigenic maps, the distances between sera and viruses are not limited to the conventional three dimensions normally visualized. However, the visualization of these data fits well in two dimensions.

#### Statistical analysis.

Statistical analysis was undertaken using Spearman’s rank correlation coefficient, Fisher’s exact test, Kruskall–Wallis test and Student’s *t*-test (significant, *P*<0.05; highly significant, *P*<0.001). Exact *P* values for the Kruskall–Wallis test, Fisher’s exact test and the Spearman’s rank correlation coefficient were calculated using StatXact software (StatXact 8 Statistical software for Exact Non-parametric Inference; Cytel Software Corporation).

#### Ethical review of human work.

This manuscript has been approved for publication by the Veterinary Laboratories Agency Ethics Committee, and the participants gave their signed consent to the work.

## Supplementary Material

Supplementary material
