# Supplementary material for: Flavivirus-induced antibody cross-reactivity
Source: J Gen Virol. 2011 Dec;92(Pt 12):2821–9. doi: 10.1099/vir.0.031641-0 (PMC3352572; doi:10.1099/vir.0.031641-0)
Supplement: Supplementary material [file supp_92_12_2821__index.html]

Flavivirus-induced antibody cross-reactivity — Supplementary Tables 

# Flavivirus-induced antibody cross-reactivity

## 

### Flavivirus-induced antibody cross-reactivity, by K. L. Mansfield, D. L. Horton, N. Johnson, L. Li, A. D. T. Barrett, D. J. Smith, S. E. Galbraith, T. Solomon and A. R. Fooks

*Journal of General Virology* vol. **92**, part 12, pp. 2821 - 2829

**Supplementary Table S1.** Neutralization results for sera obtained from a flavivirus-vaccinated human cohort

**Supplementary Table S2.** Details of viruses used in PRNTs

**Supplementary Table S3.** Neutralization titres and target distances for selected sera   
  
 [Single PDF file]  (25 KB)
