# Supplementary material for: Flavivirus-induced antibody cross-reactivity
Source: J Gen Virol. 2011 Dec;92(Pt 12):2821–9. doi: 10.1099/vir.0.031641-0 (PMC3352572; doi:10.1099/vir.0.031641-0)
Supplement: Supplementary material [file supp_92.12.2821_Supplementary_Tables.pdf]

Supplementary Table S1. Neutralization results for sera obtained from a flavivirus-vaccinated human cohort

Neg, Negative; ND, not determined.

Gender	Vaccinations	Neutralizing antibody titre ($PRNT_{50}$)							
		Sero-conversion				Cross-neutralization			
		YFV (17D)	YFV (Asibi)	TBEV	JEV	LIV	DENV-2	WNV	MVEV
M	TBEV, JEV	Neg	Neg	1:10	1:20	1:10	Neg	Neg	Neg
M	TBEV, JEV	Neg	Neg	1:10	Neg	1:10	1:20	Neg	Neg
M	TBEV, JEV	Neg	Neg	Neg	1:10	Neg	Neg	Neg	Neg
M	TBEV, JEV	Neg	Neg	1:10	1:10	1:10	Neg	Neg	Neg
M	TBEV, JEV	Neg	Neg	ND	1:20	ND	ND	Neg	Neg
M	TBEV, JEV	ND	ND	1:160	1:160	1:40	Neg	Neg	Neg
F	TBEV, JEV	Neg	Neg	Neg	1:160	1:10	Neg	Neg	Neg
F	TBEV, JEV	Neg	Neg	Neg	1:80	ND	1:20	Neg	Neg
F	TBEV, JEV	Neg	Neg	1:10	1:40	1:20	Neg	Neg	Neg
F	TBEV, JEV	Neg	Neg	Neg	1:80	1:10	Neg	Neg	Neg
F	TBEV, JEV	Neg	Neg	1:10	1:40	Neg	1:20	Neg	Neg
F	TBEV, JEV	Neg	Neg	1:10	1:20	Neg	1:40	Neg	Neg
F	TBEV, JEV	Neg	Neg	1:20	1:40	1:10	Neg	Neg	Neg
F	TBEV, JEV	Neg	Neg	1:40	1:160	1:10	Neg	1:20	Neg

Mansfield, K. L., Horton, D. L., Johnson, N., Li, L., Barrett, A. D. T., Smith, D. J., Galbraith, S. E., Solomon, T. and Fooks, A. R. (2011). Flavivirus-induced antibody cross-reactivity. *J Gen Virol* **92**, 2821–2829.

M	TBEV, JEV, YFV	Neg	Neg	Neg	1:40	Neg	Neg	1:10	Neg
M	TBEV, JEV, YFV	1:40	1:80	1:20	1:40	1:10	1:20	1:40	Neg
M	TBEV, JEV, YFV	1:80	1:80	Neg	1:10	Neg	1:20	1:10	Neg
M	TBEV, JEV, YFV	1:80	1:80	Neg	Neg	ND	Neg	Neg	Neg
M	TBEV, JEV, YFV	1:40	1:80	Neg	1:10	1:10	Neg	Neg	Neg
M	TBEV, JEV, YFV	1:20	1:20	Neg	1:160	Neg	1:20	1:160	Neg
M	TBEV, JEV, YFV	1:40	1:40	1:10	1:80	Neg	Neg	Neg	Neg
M	TBEV, JEV, YFV	1:40	1:80	1:160	1:80	1:40	1:80	1:80	Neg
F	TBEV, JEV, YFV	1:80	1:160	ND	1:160	1:10	1:20	1:80	Neg
F	TBEV, JEV, YFV	1:80	1:320	1:20	Neg	1:20	Neg	Neg	Neg
F	TBEV, JEV, YFV	Neg	Neg	1:20	1:160	1:10	Neg	Neg	Neg

Mansfield, K. L., Horton, D. L., Johnson, N., Li, L., Barrett, A. D. T., Smith, D. J., Galbraith, S. E., Solomon, T. and Fooks, A. R. (2011). Flavivirus-induced antibody cross-reactivity. *J Gen Virol* **92**, 2821–2829.

M	Unknown	Neg	Neg	1:20	1:40	1:10	Neg	Neg	Neg
M	Unknown	ND	ND	ND	1:80	Neg	ND	1:10	Neg
F	Unknown	Neg	1:40	1:1280	1:160	1:1280	1:40	1:40	Neg

Supplementary Table S2. Details of viruses used in PRNTs

Virus	Original ID	Species	Location	Date
West Nile virus (WNV) – lineage 1	DAK ArB310	Mosquito	Central Africa	1967
Louping ill virus (LIV)	Louping ill 3/1	Sheep	Oban, Scotland	1962
Tick-borne encephalitis virus (TBEV)	Neudoerfl H2J	Tick (*Ixodes ricinus)*	Austria	1950s
Japanese encephalitis virus (JEV)	P3	Mosquito	China	1949
Murray Valley encephalitis virus (MVEV)	H102/94	Unknown	Unknown	Unknown
Dengue-2 (DENV-2)	New Guinea C	Human	New Guinea	1944
Yellow fever virus (YFV)	Asibi	Human	Ghana	1927

Mansfield, K. L., Horton, D. L., Johnson, N., Li, L., Barrett, A. D. T., Smith, D. J., Galbraith, S. E., Solomon, T. and Fooks, A. R. (2011). Flavivirus-induced antibody cross-reactivity. *J Gen Virol* **92**, 2821–2829.

YFV	17D	Vaccine strain derived from YFV Asibi, passage 240

Supplementary Table S3. Neutralization titres and target distances for selected sera

ED_{50}, Reciprocal 50% end-point dilutions; TD, target distance (described in Methods) used for Fig. 3; *, no distance.

**Serum**	**TBEV**		**JEV**		**YF 17D**		**YF Asibi**		**WNV**		**LIV**		**DENV-2**	
	**ED_{50}**	**TD**	**ED_{50}**	**TD**	**ED_{50}**	**TD**	**ED_{50}**	**TD**	**ED_{50}**	**TD**	**ED_{50}**	**TD**	**ED_{50}**	**TD**
**1**	10	7.0	20	6.0	<10	>7.0	<10	>7.0	<10	>7.0	10	7.0	<10	>7.0
**2**	10	7.0	<10	>7.0	<10	>7.0	<10	>7.0	<10	>7.0	10	7.0	20	6.0
**4**	10	7.0	10	7.0	<10	>7.0	<10	>7.0	<10	>7.0	10	7.0	<10	>7.0
**6**	160	3.0	160	3.0	<10	>7.0	<10	>7.0	<10	>7.0	40	5.0	<10	>7.0
**9**	10	7.0	40	5.0	<10	>7.0	<10	>7.0	<10	>7.0	20	6.0	10	>7.0
**11**	10	7.0	40	5.0	<10	>7.0	<10	>7.0	<10	>7.0	<10	>7.0	20	6.0
**12**	10	7.0	20	6.0	<10	>7.0	<10	>7.0	<10	>7.0	<10	>7.0	40	5.0
**13**	20	6.0	40	5.0	<10	>7.0	<10	>7.0	<10	>7.0	10	7.0	<10	>7.0
**14**	40	5.0	160	3.0	<10	>7.0	<10	>7.0	20	6.0	10	7.0	<10	>7.0
**16**	20	6.0	40	5.0	40	5.0	80	4.0	40	5.0	10	7.0	20	6.0
**17**	<10	>7.0	10	7.0	80	4.0	80	4.0	10	7.0	<10	>7.0	20	6.0
**19**	<10	>7.0	10	7.0	40	5.0	80	4.0	<10	>7.0	10	7.0	<10	>7.0

Mansfield, K. L., Horton, D. L., Johnson, N., Li, L., Barrett, A. D. T., Smith, D. J., Galbraith, S. E., Solomon, T. and Fooks, A. R. (2011). Flavivirus-induced antibody cross-reactivity. *J Gen Virol* **92**, 2821–2829.

**22**	160	3.0	80	4.0	40	5.0	80	4.0	80	4.0	40	5.0	80	4.0
**23**	*	*	160	3.0	80	4.0	160	3.0	80	4.0	10	7.0	20	6.0
**24**	20	6.0	<10	>7.0	80	4.0	320	2.0	<10	>7.0	20	6.0	<10	>7.0
**25**	20	6.0	160	3.0	<10	>7.0	<10	>7.0	<10	>7.0	10	7.0	<10	>7.0
**26**	20	6.0	40	5.0	<10	>7.0	<10	>7.0	<10	>7.0	10	7.0	<10	>7.0

Mansfield, K. L., Horton, D. L., Johnson, N., Li, L., Barrett, A. D. T., Smith, D. J., Galbraith, S. E., Solomon, T. and Fooks, A. R. (2011). Flavivirus-induced antibody cross-reactivity. *J Gen Virol* **92**, 2821–2829.
